# High Mutation Frequency and Significant Population Differentiation in Papaya Ringspot Virus-W Isolates

**DOI:** 10.3390/pathogens10101278

**Published:** 2021-10-04

**Authors:** Vivek Khanal, Akhtar Ali

**Affiliations:** Department of Biological Science, The University of Tulsa, Tulsa, OK 74104, USA; vik684@utulsa.edu

**Keywords:** selection pressure, mutation, coat protein, polymorphism, substitution

## Abstract

A total of 101 papaya ringspot virus-W (PRSV-W) isolates were collected from five different cucurbit hosts in six counties of Oklahoma during the 2016–2018 growing seasons. The coat protein (CP) coding region of these isolates was amplified by reverse transcription-polymerase chain reaction, and 370 clones (3–5 clones/isolate) were sequenced. Phylogenetic analysis revealed three phylogroups while host, location, and collection time of isolates had minimal impact on grouping pattern. When CP gene sequences of these isolates were compared with sequences of published PRSV isolates (both P and W strains), they clustered into four phylogroups based on geographical location. Oklahoman PRSV-W isolates formed one of the four distinct major phylogroups. The permutation-based tests, including Ks, Ks *, Z *, Snn, and neutrality tests, indicated significant genetic differentiation and polymorphisms among PRSV-W populations in Oklahoma. The selection analysis confirmed that the CP gene is undergoing purifying selection. The mutation frequencies among all PRSV-W isolates were within the range of 1 × 10^−3^. The substitution mutations in 370 clones of PRSV-W isolates showed a high proportion of transition mutations, which gave rise to higher GC content. The N-terminal region of the CP gene mostly contained the variable sites with numerous mutational hotspots, while the core region was highly conserved.

## 1. Introduction

Papaya ringspot virus (PRSV), a single-stranded, positive-sense RNA virus, belongs to the family *Potyviridae* and genus *Potyvirus*. The virus is primarily grouped into two serologically indistinguishable strains: papaya-infecting type (PRSV-P) and cucurbit-infecting type (PRSV-W) [1]. The host range of PRSV-W is limited to Chenopodiaceae and Cucurbitaceae, while PRSV-P can infect plants in the papaya family (Caricaceae) as well [1,2,3]. The site-directed mutagenesis study in recombinant viruses showed that lysine amino acid (aa) at position 27 in NIa-Pro determines the host specificity in PRSV-P, as a single aa change at that position from lysine to aspartic acid can change the host range of PRSV-P to non-papaya-infecting [4].

PRSV particles are non-enveloped, flexuous, and filamentous rods about 680–900 nm in length and 12–15 nm in diameter. They are transmitted by several aphid species in a non-persistent, non-circulative manner [5,6]. The virus can be mechanically inoculated, while no seed transmission has been reported. Similar to other potyviruses, PRSV has a linear single-stranded and positive-sense RNA (+ssRNA) genome that is approximately 10.3 kb. The PRSV genome comprises a 5ʹ untranslated region, a single open reading frame (ORF) that codes for a major polyprotein, and a 3’ untranslated region. The polyprotein is proteolytically processed by virus-encoded proteases into 10 mature proteins: P1 (63k), helper component protease (Hc-Pro, 52k), P3 (46k), 6K1 (6k), cylindrical inclusion (CI, 72k), 6K2 (6k), nuclear inclusion protein a-virus protein genome linked (NIa-Vpg, 27k), nuclear inclusion protein a-protease (NIa-Pro 21k), nuclear inclusion protein b (NIb, 59k), and coat protein (CP, 36K) [7]. A small protein called pretty interesting potyvirus ORF (PIPO) is synthesized by an additional ORF in the P3 region [8].

The CP gene of potyviruses, including PRSV, is located at the 3’ terminal end of the viral genome and encapsidates the RNA genome of the virus. The CP of potyviruses is a multifunctional protein and has a significant role in the viral life cycle. For instance, it is involved in aphid transmission in association with HC-Pro [9], cell-to-cell and systemic movement [10], virus assembly [7], and host adaptation [11]. Classification of potyviruses based on the CP gene was quite common until the mid-2000s as it is considered the most conserved protein among potyviruses. CP is the only structural protein in potyviruses, and its multiple subunits form a protective coat for the RNA genome. The cleavage motif for CP and Nib region in PRSV-W is VFHQ/S [12].

At least 94 viruses from 17 different families, including *Potyviridae,* have been reported to infect cucurbit crops [13], while 15 of those belong to the genus *Potyvirus* [6] PRSV-W is one of the major viruses infecting cucurbits that cause substantial yield losses of cucurbits worldwide [13,14,15,16,17,18,19,20]. PRSV-W induces a wide range of symptoms in different cucurbit crops, which includes mosaics, mottling, stunting, vein clearing, shoestrings on leaves, ringspots, and streaks on fruit stems and petioles, thereby reducing both quality and quantity of fruit production [13].

The highly error-prone, RNA-dependent RNA Polymerase (RdRp) of RNA viruses, including PRSV-W, lack proofreading ability, enabling them to generate a large pool of genetically distinct sequences (often referred to as a ‘mutant swarm’) in a short generation time compatible with the concept of viral quasispecies [21]. These attributes contribute to high levels of genetic diversity, the ability to adapt to changing environments, including new hosts, and to evade host resistance [22]. Numerous molecular studies have been conducted in recent decades on ecology, etiology, pathogenesis, molecular biology, diversity, evolution, and control strategies of PRSV-P, but very few about PRSV-W. Thus, further investigation of the evolution of the viral population is important to deduce reliable diagnostic tools and effective management strategies for combating PRSV-W.

Previously, an evolutionary study was conducted on 64 PRSV-W isolates from watermelon in Oklahoma during a single growing season with a limited area of sampling [12]. In this study, we performed a comprehensive evaluation of population differentiation and genetic diversity among >100 PRSV-W isolates from six counties of Oklahoma (Figure S1) during three growing seasons. We hypothesized that since the virus sequences were sampled within a short span of time, there would be strong purifying selection, and the population would consist of a mutant cloud or mutant swarm. Our other hypothesis was that the phylogenetic grouping of the isolates would be influenced by geographical location, hosts, and year of collection. We also evaluated mutation frequency and its pattern within individual clones of PRSV-W isolates collected from different regions of Oklahoma.

## 2. Results

### 2.1. PRSV-W Isolates and Confirmation by RT-PCR 

A total of 101 PRSV-W isolates were collected from six counties (Blaine, Caddo, Cimarron, McCurtain, Muskogee, and Tulsa) of Oklahoma from five different hosts (cantaloupe, cucumber, pumpkin, squash, and watermelon) during the 2016–2018 growing seasons. The complete CP gene of these isolates was amplified by reverse transcription-polymerase chain reaction (RT-PCR), and the expected size DNA bands were gel-purified. PRSV-W positive samples were also checked by RT-PCR for mixed infection with specific primers of watermelon mosaic virus (WMV) and zucchini yellow mosaic virus (ZYMV) [13]. Fifty-five of these 101 PRSV-W had mixed infection with WMV, ZYMV, or both. 

### 2.2. CP Gene Sequence Analysis

A total of 370 recombinant clones (3–5 clones from each isolate) were sequenced from 101 PRSV-W isolates in this study. The complete CP gene sequence of all PRSV-W clones was 864bp, which translated into 287 aa residues except one clone, which had 24 nucleotides (nt) deletion at the N-terminal region of the CP gene. The nt identity among consensus CP sequences of 101 PRSV-W isolates from this study ranged from 96 to 100%, and aa identity was 98–100%. The 165 Oklahoman isolates (101 from this study and 64 isolates from the previous study [12]) had 86–98% nt identity and 87–98% aa identity with other isolates from around the globe (Table 1).

### 2.3. Phylogenetic Relationship among PRSV-W Populations

**Figure 1:**
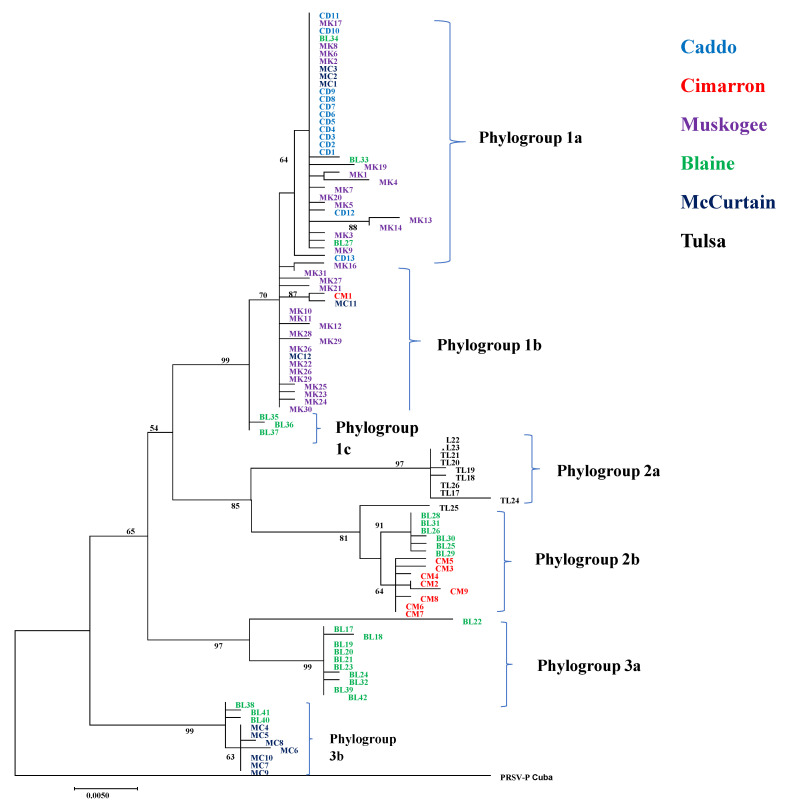
Maximum likelihood (ML) phylogenetic tree constructed in MEGA 7 using general time-reversible (GTR) model for coat protein gene of 101 PRSV-W isolates in this study. Name of the isolates is shown at the tip of the tree. The bootstrap values >50 are shown at the respective nodes. The phylogenetic grouping is shown at right side of the phylogenetic tree. The PRSV-P isolate from Cuba was used as an outgroup.

The maximum likelihood (ML) phylogenetic tree constructed from the CP gene with 101 PRSV-W isolates in this study showed three distinct phylogroups circulating in cucurbit fields of Oklahoma (Figure 1). Phylogroup 1 was further divided into three subgroups. Subgroup 1a included 28 isolates that belong to four counties (Caddo, McCurtain, Muskogee, and Blaine). Subgroup 1b had 20 isolates from three counties (Muskogee, McCurtain, and Cimarron). Subgroup 1c had three isolates from Blaine County. Phylogroup 2 consisted of two subgroups; 2a consisted of 9 isolates collected from Tulsa County, and 2b of 15 isolates from three counties (Blaine, Cimarron, and Tulsa). Phylogroup 3 consisted of two subgroups; 3a consisted of 11 isolates from Blaine, and 3b contained 10 isolates from two counties (Blaine and McCurtain). The ML phylogenetic tree made with sequences from this study and 64 sequences from the previous study [12] also showed a similar pattern (Supplementary Figure S2). Although that study included PRSV-W isolates from two counties (Atoka and Jefferson) not included in this study, they also clustered together with isolates from other counties.

**Figure 2:**
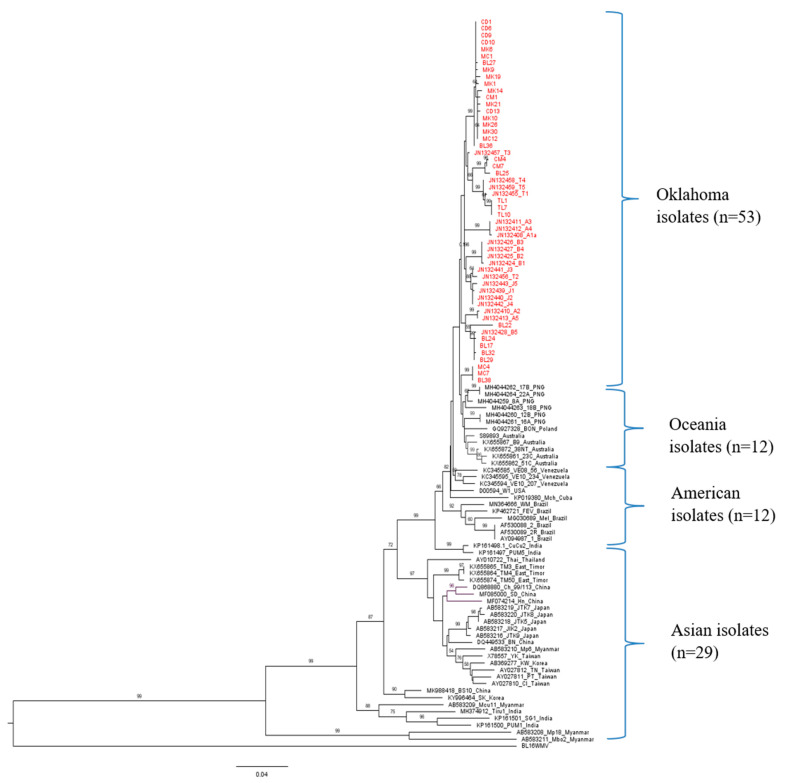
Maximum likelihood (ML) phylogenetic tree constructed in MEGA 7 using general time-reversible (GTR) model for coat protein gene of 106 PRSV-W isolates (33 from this study and 73 from GenBank). The isolates from Oklahoma are shown in red color. GenBank accession number, isolate name, and country of origin are shown on each node (GenBank accession number is not shown for isolates from this study). The bootstrap values >50 are shown at the respective nodes. The phylogenetic grouping is shown on right side of the phylogenetic tree. The complete coat protein sequence of WMV isolated from Blaine County was used as an outgroup.

Selected PRSV-W isolates: 33 out of 101 from this study and 73 retrieved from GenBank were used to deduce the ML phylogenetic tree with the CP gene of WMV as an outgroup (Figure 2). The selection of sequences from Oklahoma for global phylogenetic analysis was based on their phylogenetic position within Oklahoma isolates. Four distinct clusters of PRSV-W isolates were observed, loosely based on geographical location. All Asian isolates were grouped into Cluster 1 (C1), while Cluster 2 (C2) contained both North and South American isolates, except isolates from Oklahoma. Cluster 3 (C3) included all the isolates from Oceania, while Cluster 4 (C4) contained all the isolates from Oklahoma. The two isolates from Europe clustered together with American (French isolate) and Oceania isolates (Polish isolate). In addition, another phylogenetic tree was constructed using 148 PRSV isolates that contained both PRSV strains (P and W), including 42 isolates of PRSV-P retrieved from GenBank (Supplementary Figure S3). The Oklahoma isolates are again grouped in a separate cluster (C4). The clustering pattern was similar to PRSV-W, with Asian isolates in C1, other American isolates in C2, and Oceanian isolates in C3.

### 2.4. Genetic Variation among PRSV-W Populations

The overall genetic diversity (d) in the CP gene of PRSV-W isolates from Oklahoma was 0.020; the average number of nt differences (K) was 16.09, and nt diversity (π) was 0.019 (Table 2). PRSV-W isolates from Caddo County were the least diverse (d = 0.001, K = 2.07, π = 0.002), while isolates from Blaine County were the most diverse (d = 0.023, K = 18.02, π = 0.022). The estimates of evolutionary distances in the CP gene between PRSV-W isolates from different counties of Oklahoma showed that populations from Caddo County and Muskogee County were least distant (d = 0.003), and populations between McCurtain County and Tulsa County were most distant (d = 0.036) (Supplementary Table S3). The estimates of evolutionary divergence in the CP gene among PRSV-W isolates within different hosts were fairly consistent with genetic distance and nucleotide diversity in the range of 0.15 < d < 0.24 and 0.015 < π < 0.02, respectively. Similarly, the divergence in the CP gene between PRSV-W populations from different hosts was also close to the mean overall distance (Supplementary Table S3). The genetic distances within the CP gene of PRSV-W populations collected in 2016, 2017, and 2018 were 0.012, 0.013, and 0.024, respectively. Similarly, the nt diversities in the CP gene among PRSV-W in 2016, 2017, and 2018 were 0.013, 0.014, and 0.015, respectively. The CP gene of PRSV-W populations collected between 2016 and 2017 had a genetic distance of 0.014; between 2016 and 2018 was 0.022, while between 2017 and 2018 was 0.021. The mean genetic distances (and nt diversity) within PRSV-W populations in phylogroups 1, 2, and 3 were 0.003 (π = 0.04), 0.015 (π = 0.015), and 0.019 (π = 0.016), respectively (Table 2). The divergence between phylogroup 1 and 2 was 0.027, phylogroup 1 and 3 was 0.024, and between phylogroup 2 and 3 was 0.036 (Supplementary Table S3). The haplotype diversity (Hd) for each of the aforementioned groups was high, with an overall Hd of 0.98 (Table 2).

For further analysis, we used 33 selected CP gene sequences from this study, 73 PRSV-W isolates, and 42 PRSV-P isolates from GenBank to calculate genetic diversity among PRSV global isolates (Table 3). The overall mean genetic diversity in the CP gene of all selected PRSV-W isolates (n = 106) was 0.071, and all PRSV isolates (n = 148) was 0.085 (Table 3). The overall average number of nt differences (K) between PRSV-W isolates was 51.71, and nt diversity (π) was 0.07 (Table 3). The average number of nt differences and nt diversity was higher (K = 61.89, π = 0.08) in the overall PRSV group (n = 148). Between different phylogroups of PRSV, Asian isolates were most diverse (d = 0.11, K = 74.95, π = 0.09) and Oceania isolates were least diverse (d = 0.02, K = 18.27, π = 0.021). The haplotype diversity for each of these groups was >0.95, with an overall Hd of 0.99 (Table 3 and Supplementary Table S4).

### 2.5. Population Differentiation within PRSV-W Populations

The total genetic variability in a population compared to the total population and gene flow estimated from F statistics (*Fst*) showed that there is infrequent gene flow of PRSV-W between different counties (*Fst* >0.33) except between Blaine and McCurtain counties (*Fst*=0.24) and Caddo and Muskogee (*Fst* =0.14), where gene flow was frequent (Table 4). Similarly, the Nm value for all pairs of counties was <1 except CD and MK (Nm = 1.60). The *Fst* values for all pairs of PRSV-W hosts were <0.33, with only three pairs of hosts (cucumber and pumpkin, cucumber and watermelon, and pumpkin and squash) having an Nm value <1. The three phylogroups of PRSV-W within this study had infrequent gene flow and distinct population differentiation. However, the population of PRSV-W within the three years of the collection had frequent gene flow (*Fst* < 0.33, Nm > 1) but were genetically distinct based on other statistical tests. The P values for all permutation-based tests Ks, Ks*, Z*, and Snn were <0.01 for almost all the population groups showing significant genetic differentiation (Supplementary Table S5). The values of all three neutrality tests (Fu and Li’s D and F and Tajima’s D) for all population groups were negative and occasionally significant (Supplementary Table S6). The negative values on neutrality tests indicate polymorphisms within PRSV-W populations.

Population differentiation analyses were performed for different PRSV populations based on their phylogenetic groups and geographical locations. The *Fst* value for PRSV-W populations between Asia and other parts was >0.33, indicating an absence of gene flow between these groups (Table 5). The significant *Fst* value was also supported by low Nm values. All other PRSV-W groups had an *Fst* value <0.33. However, when PRSV-P isolates were added to the study, the *Fst* value for all groups dropped below 0.33. All the permutation-based tests confirmed that there was distinct genetic differentiation between these groups (Supplementary Table S7). All the phylogroups from various parts of the world had non-significant negative values on three neutrality tests (Supplementary Table S8).

### 2.6. Mutation Frequency 

The overall mutation frequency (f) among 370 sequences obtained from 101 PRSV-W isolates was 1.18 × 10^−3^. The mutation frequency within a single infection of PRSV-W isolates was 1.22 × 10^−3,^ and that of PRSV-W mixed with one or more viruses was 1.15 × 10^−3^. The average mutation frequencies for each county, host, and collection year in a single and mixed population were determined and compared (Table 6, Table 7 and Table 8). The average mutation frequency in different counties ranged from 0.79 × 10^−3^ (McCurtain County) to 1.37 × 10^−3^ (Tulsa County). The mutation frequencies of the virus in different counties were generally higher in single infections than in mixed infections, except in Blaine County (Table 6). Similarly, mutation frequencies in different hosts ranged from 0.98 × 10^−3^ (squash) to 1.5 × 10^−3^ (cucumber). The mutation frequencies in single and mixed infections were fairly close to the overall mutation frequency within the same host (Table 7). The average mutation frequencies of PRSV-W populations in 2016 was 1.23 × 10^−3^, which increased slightly in 2017 (1.3 × 10^−3^) and was down to 1.07 × 10^−3^ in 2018. As in the case of different hosts, mutation frequencies in single and mixed infections were similar to overall mutation frequency in all three years. The average mutation frequency (1.60 × 10^−3^) in the C-terminal region of the CP gene was highest among the three regions of the gene, followed by the N-terminal (1.10 × 10^−3^) and core region (1.01 × 10^−3^). 

### 2.7. Selection Pressure Analysis

In almost all aforementioned populations, the number of non-synonymous mutations exceeded synonymous mutations. However, dN/dS of value did not equal or exceed 1 in any of the populations, indicating negative (purifying) selection. The selection analysis using four independent tests (FUBAR, FEL, MEME, and SLAC) available in the Datamonkey server showed a number of codons undergoing negative (purifying) selection (Table 9). While FEL, MEME, and SLAC did not show evidence of positive selection within the CP gene of Oklahoman isolates, FUBAR analysis showed two codons at positions 76 and 172 undergoing positive selection. All four tests showed the presence of a few positively selected codons in PRSV global populations. At least two tests showed evidence of positive selection in codon positions 14, 16, 43, 48, 82, 90, and 256.

### 2.8. Mutational Pattern 

**Figure 3:**
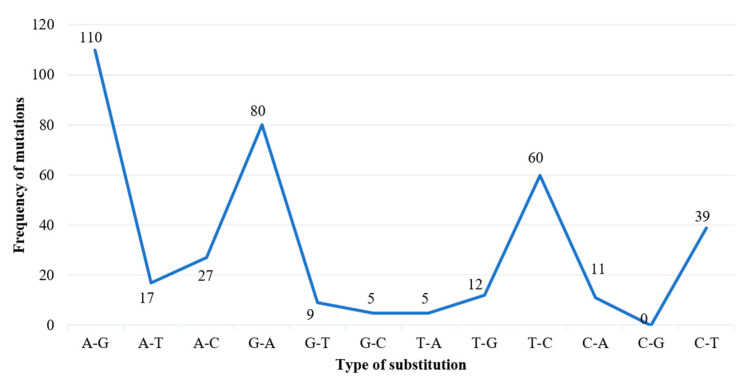
Nature of substitution mutations observed in 370 recombinant clones from 101 PRSV-W isolates. The Y-axis shows the frequency of nucleotide substitutions observed within the CP gene of PRSV-W isolates, and X-axis shows the type of nucleotide substitution.

**Figure 4:**
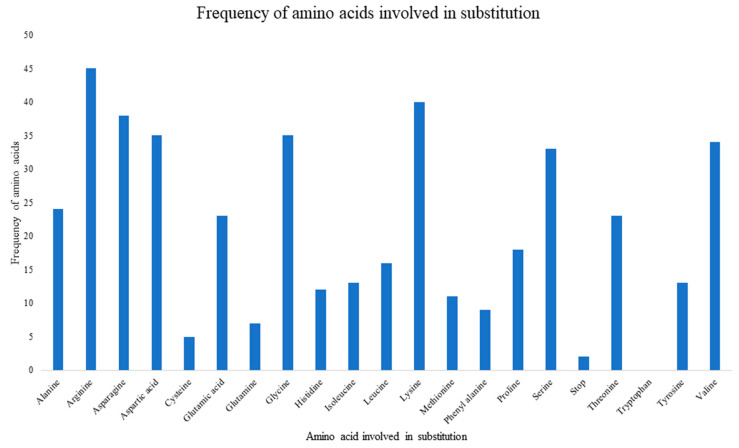
Bar chart showing frequency of amino acids involved in substitution mutations within PRSV-W coat protein gene. The total number of amino acids and stop codons involved was 436, as there were 218 non-synonymous mutations.

**Figure 5:**
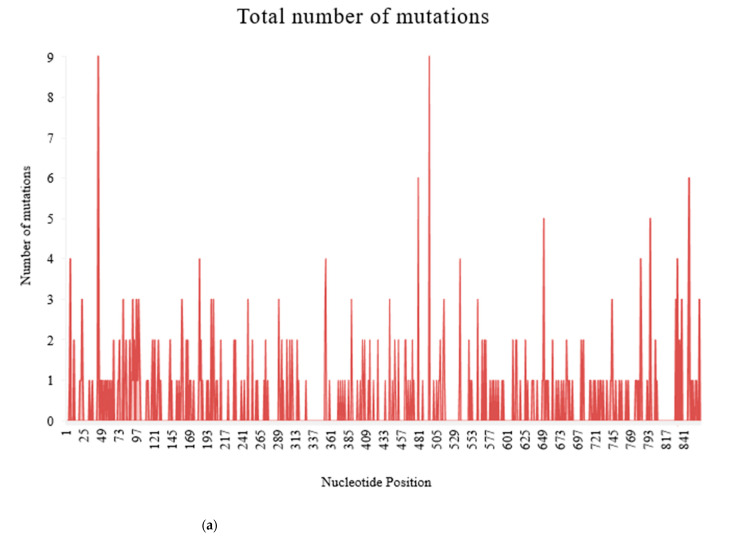

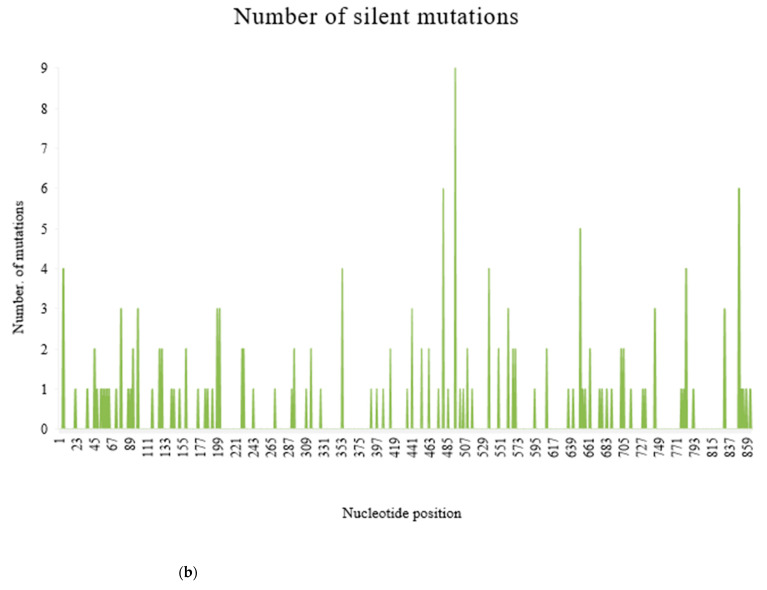

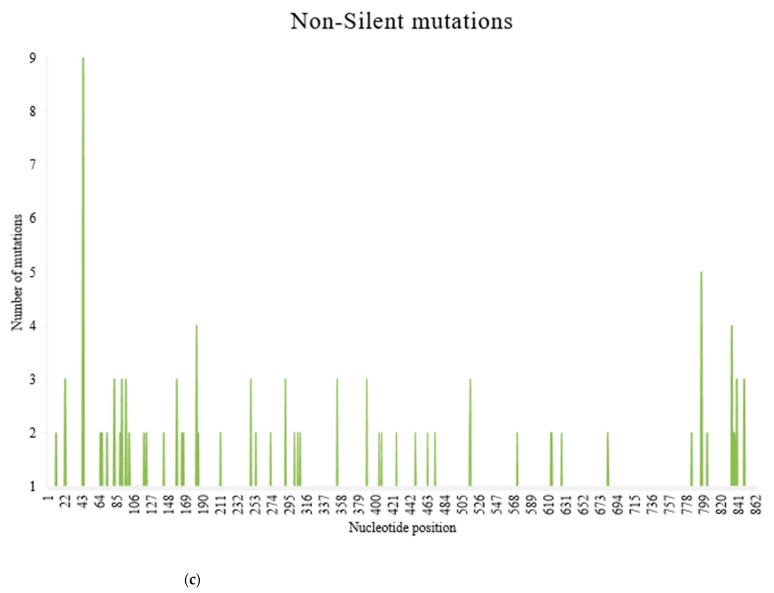
Frequency of substitution mutations at different nucleotide positions within 370 recombinant clones from 101 PRSV-W isolates. Total of 864 sites was present, corresponding to each nucleotide in the coat protein region. (**a**) Frequency of total substitution mutations. (**b**) Frequency of silent mutations. (**c**) Frequency of non-silent mutations.

There were 376 substitution mutations among 370 clones of 101 PRSV-W isolates (Figure 3). Transition (293, 77.93%) were three-fold higher than transversion (82, 22.07%). A substitution from Adenine (A) to Guanine (G) was most common (110, 29.26%), followed by its reverse substitution G–A (80, 21.28%). Another transition substitution, Thymine (T) to Cytosine (C) (60, 15.96%) and its reverse substitution C–T (39 (10.37%), were also frequent. All transversion had a frequency <8%, with substitution from A to T being most of the lot (27, 7.18%), while substitution from C to G was null. There was one clone of PRSV-W from Caddo County collected in 2016 (CD-2) with a deletion of 24 nt from positions 57–80. Among those 376 substitutions, 218 were non-synonymous and had 77 combinations of aa substitutions. Twenty-six of those aa substitutions were only observed once, and twenty-four were only observed two times. The most common aa substitution among the CP gene of the PRSV-W population involved Lysine to Arginine, with a frequency of 14 out of 218 non-synonymous substitutions (6.34%) (Table 10). Similarly, a change of aa from Asparagine to Aspartic acid was observed 12 times. The aa changes from Arginine to Lysine (9 times), Alanine to Valine, Glutamic acid to Glycine, and Leucine to Proline (8 times) were also frequent. There were also two mutations leading to the stop codon. The top 5 aa involved in non-synonymous substitutions were Arginine, Lysine, Asparagine, Aspartic acid, and Glycine (Figure 4). There were no mutations observed involving tryptophan. A total of 231 sites (nt positions) had at least one substitution. The number of non-synonymous sites was 145, and that of synonymous sites was 86. Substitution on 12 of these sites occurred >3 times, 20 sites occurred 3 times, 53 sites occurred 2 times, and 146 sites occurred only once. nt positions 44 and 495 of the CP genome had the highest number of nt changes (9), followed by positions 480 and 849, with 6 nt changes (Figure 5a–c). Among these 4 nt changes, the only substitution on position 44 (aa position 15) was non-silent. The other frequent non-synonymous sites were aa positions 266 (5 changes), 61, and 278 (4 changes).

The entire CP gene was subdivided into three regions: N-terminal, core, and C- terminal. The N-terminal region included 1–195 nt (1–65aa), the core region included 196–654 nt (66–218aa) and the C-terminal included 655–864 nt (219–287aa). Out of 287 aa in the CP gene, 229 aa sites were conserved among all PRSV-W isolates obtained in this study from Oklahoma. The core region was highly conserved as nearly 78% of the sites were completely conserved (without any mutations), in addition to 8.5% of the sites with silent mutations (Table 11). Conversely, the N region had only 62.6% of the sites, which were conserved with a higher number of mutational hotspots in the region. The mean genetic diversity was also highest (0.019) in the N-region. Most of the frequent non-silent mutations were observed in either the N-terminal or C-terminal of the CP gene. The most conserved site within CP was at the core region, with 29 consecutively conserved aa sequences from positions 121 to 149 among the consensus sequences from this study.

## 3. Discussion

This study confirms our first hypothesis that there is a strong purifying selection within the PRSV-W populations. The selection is likely acting on removing deleterious mutations caused by error-prone replication. The high mutation frequency within all PRSV-W populations from Oklahoma confirms that the population exists as mutant clouds compatible with the quasispecies concept. The PRSV-W populations within this study were collected in a relatively quick time frame, which makes high mutant clouds the likely outcome [23]. Additionally, these mutation frequencies were remarkably consistent in all the populations (based on geography, hosts, collection years, and single/mixed infections) within the range of 10^−3^. Mutations are the major driving forces for monopartite RNA virus variation in addition to recombination. The error rate of RNA replication ranges from 10^−3^–10^−5^ base per copying cycle giving rise to high diversity within populations due to large mutant clouds. The high mutation rates reflect an evolutionary strategy as these mutant clouds usually work in favor of viruses for adaptation during environmental stress [24,25]. The majority of the mutation events in this study were substitutions, and rarely any insertion/deletions (indels) were found. The indel mutations are usually rare but are lethal in most cases [26]. Due to the abundance of deleterious mutations, the level of dN is generally higher in closely related sequences compared to distantly related sequences [27], which explains slightly higher purifying selection in Oklahoma isolates compared to global isolates.

The replication rates of most RNA viruses are swift, so they are able to reach exceptionally large population sizes within a brief period of time [26]. However, this large population size is not, in fact, an effective population size, as a substantial part of this population consists of mutants that will not pass to the next generation [25]. The genetic bottleneck reduces the population size below a threshold level to facilitate the transmission of fittest variants, thereby limiting the size of the effective population [28]. The genetic bottleneck usually is the product of the biology of the vector and its feeding habit and can also occur at different moments of the viral life cycle, such as virus movement between plant cells during systemic infection and horizontal transmission [29,30,31,32]. In addition, the purifying selection helps viruses maintain genetic stability by eliminating less-fit mutants with deleterious effects [33]. The genetic variations due to mutation are also structured by gene flow [34]. The gene flow among different hosts, geographical regions, and different parts of the same plant helps in shaping the global genetic diversity [35]. The low level of long-distance movement or gene flow might be the reason behind the non-uniform and variable viral populations in this study. However, this low level of gene flow was enough to accommodate variants from different phylogroups occurring in the same geographical area. 

Utmost care was taken to reduce the mutations during RT-PCR steps by employing a number of strategies: high-fidelity reverse transcriptase and Taq polymerase were used, the number of PCR cycles was limited to 25, and any mutations found in only one direction of Sanger sequencing were not considered. Despite these precautions, there is a chance that some of these mutations might be due to experimental error. Similar to the study conducted by Simmons et al. [36], we calculated the highest possible number of erroneous mutations due to RT-PCR. The total number of possible mutations due to RT was ∼9 (2.9 × 10^−5^ mutations × 864 sites × 370 clones), and PCR was ∼7 (2.28 × 10^−5^ mutations × 864 sites × 370 clones), which adds to a total of ∼16 mutations. Even if we deduct these (possible) artifact mutations (16) from the total number of mutations (376) observed in the study, the mutation frequency remains in a similar range. However, the actual erroneous mutations due to experimental error might be significantly less than these calculated values due to the aforementioned experimental considerations. 

The PRSV-W isolates collected from the same county, host, and growing season were grouped in different phylogroups, while isolates collected from different counties were grouped in the same phylogroup. This rejects our second hypothesis that these factors play a role in phylogenetic clustering. For instance, PRSV-W isolates collected from Blaine County in a single growing season (2017) from the same host (pumpkin) grouped in three different phylogroups (Figure 1). Similarly, PRSV-W isolates from Blaine, Cimarron, and McCurtain counties collected in 2018 fell in two different phylogroups. This diversity is also well supported by the higher within-group mean evolutionary distance of PRSV-W isolates from these counties compared to others (Table 2). Some of the isolates from geographically distant locations (Muskogee and Caddo counties) even had identical sequences. In addition, isolates from two far corners of Oklahoma with more than 900 kilometers of distance were grouped together closely in the same phylogroup (phylogroup 1), irrespective of their collection year and host. The close evolutionary distance between isolates from various locations might have caused the close evolutionary relationship. The lack of geographical connectivity in phylogeny among these isolates can be attributed to different possibilities. First, all these isolates from Oklahoma might have been derived from the same most recent common ancestors (MRCA). Second, the virus or virus harboring aphids likely travels with harvested plants and fruits to various parts of the state, thereby facilitating spread in new locations. In both cases, the virus population can use the wild host as their reservoir during times other than the growing seasons of their primary hosts [13]. In addition, none of the phylogroups within Oklahoma had distinct fixed mutations in terms of nt and aa, indicating the recent common ancestry of all these populations. The anomaly was the isolates from Tulsa, which had three distinct aa changes compared to other populations at positions 44 (Alanine–Threonine), 76 (Valine–Isoleucine), and 120 (Serine–Asparagine). 

Similarly, other parameters considered in the study, viz. host and collection years, also did not have a significant effect on phylogeny. For instance, the genetic differentiation between different hosts and their diversity was not significant in all populations of PRSV, and none of the frequent mutations were observed in specific hosts. The PRSV-W virus isolates collected at different points of time (2008–2018) from the same location did not cluster according to the collection years. However, if the isolates collected in the same year fell in the same phylogroup, they tended to group together, indicating a loose association between collection time and their evolutionary fate. This is further bolstered by the fact that mutation frequencies of virus isolates collected from different hosts and in different collection years remained highly similar (Table 8 and Table 9).

The clustering pattern of global isolates showed distinct geographical clustering, with Asian, American, and Oceania isolates falling in different phylogroups. The two European isolates from France and Poland were anomalous as they were grouped with two distinct groups. More isolates from Europe are needed to evaluate if all of them cluster together with either American and Oceanian isolates or form separate clusters among themselves. The grouping pattern in the phylogeny of PRSV-W in this study is similar to recent studies [37,38], which showed that isolates from different parts of the world grouped together with one phylogroup and a few Asian isolates in different phylogroup. The PRSV isolates from other parts of the US were close to Mexican and other American isolates, as observed previously [39,40]. The distinct geographical clustering of the PRSV population based on continents shows PRSV-W populations do not have recent travel history across the continents (North and South America are referred to here as ‘Americas’). While the aforementioned (refer to the previous paragraph) factors explain geographical connectivity among virus populations in nearby locations, longer distance movement from these modes of transmission is unlikely. Similar to the clustering pattern in Oklahoma, the global isolates did not have distinct phylogenetic differentiation among hosts and collection years. 

More than 99% of the mutations observed were substitution mutations. These substitution mutations were biased towards transitions with a high proportion (>75%) of purine to purine or pyrimidine to pyrimidine nt change (Figure 4). The transition mutation biases are common in viral systems and were noted in several previous studies [41,42,43,44]. All the combinations of nt substitutions involving G and C had the mutations favoring change to these nt, thereby favoring gain of net GC content. This net gain of GC content was also observed in a study conducted by Nigam et al. [44]. In addition, the resulting aa changes from these GC-rich mutations mostly involved aa Arginine, Lysine, Asparagine, Aspartic acid, Glycine, and Alanine with either loss or gain of the net charge. Interestingly, all these aa are also disorder-promoting [45]. Conversely, the order-promoting aa, such as Tryptophan and Cysteine, were rarely involved in substitution mutations.

Mutation frequency within the N-terminal region of CP was highest, followed by the C-terminal region and core region. Although mutations were frequent in core regions, they were disproportionately silent and included less positively selected sites in comparison to N- and C-terminal regions. This further consolidates the evidence of a highly conserved core region. All but seven isolates from McCurtain County, which were collected in 2018, had a DAG motif at amino acid positions from 7 to 9 in this study. These seven isolates have Threonine instead of Alanine. The Alanine to Threonine mutation was also observed in three other global isolates of PRSV; one from the USA and two from Taiwan. In addition, few isolates had NAG, DSG, and DSA instead of DAG motif. The DAG motif, highly conserved among Potyvirus CP genes, has a significant role in virus transmission by aphids [46] and is exposed on the viral surface [47]. However, the mutation in this region has been reported in many studies with efficient aphid transmission [48,49,50,51,52,53]. The other two motifs, PTK and KITC, present in another Potyvirus gene, Hc-Pro, and their interaction with the CP gene also have a vital role in viral transmission by aphids [54,55], and these motifs could facilitate aphid transmission in the absence of the DAG motif [49]. A number of conserved motifs described in Potyvirus CP previously were present in PRSV-W populations in this study with minor or no mutations. In addition, there were more highly conserved motifs in the core region of CP, which are specific to PRSV (Table 12). These conserved motifs were present in PRSV populations regardless of the biotype and might carry some evolutionary role. Further study is desired to decode the evolutionary messages conveyed by these motifs. The presence/absence of these motifs nevertheless could be useful in diagnostic tools such as primer design.

Recombination analysis of all 101 PRSV-W isolates sequenced in this study, as well as all 165 Oklahoman PRSV-W isolates (101 isolates from this study and 64 isolates from the previous study), was conducted using RDP software. Only two recombination events were detected by two algorithms in 101 PRSV-W isolates and were not significant (data not shown). These results indicate that recombination events are not frequent in the CP-gene-coding region.

To our knowledge, this is the first study on mutational analysis within the quasispecies population of PRSV-W isolates. The present study provides a broad analysis of a wide range of PRSV-W populations isolated from diverse geographical locations of the state, host, and collection time based on the various aspects of evolution such as mutation, genetic differentiation, and phylogeny. The insights provided by this study will enhance existing knowledge of PRSV-W evolution and epidemiology and will be helpful in developing viable management strategies. Specifically, the high diversity of PRSV populations in different geographical locations and the possibility of multiple viral introductions in the same geographical location demand careful consideration towards accommodating different genetic aspects of the virus in multiple locations while developing sustainable control strategies. 

## 4. Materials and Methods

### 4.1. Sample Collection and Detection of PRSV-W

Surveys were conducted during the three growing seasons of 2016–2018 in multiple counties of Oklahoma from different cucurbit hosts, and dot immunobinding assay (DIBA) was performed against 10 different viruses [13]. Confirmation of PRSV-W was completed by RT-PCR using the primers specific for the CP gene of PRSV-W (PRSVCPF; 5ʹCTGATGATTATCAACTTGTT3ʹ, PRSVCPR; 5ʹTAAGGTGAAACAGGGTGGAG3ʹ) as described previously with minor modifications [13]. Total RNA was extracted by the Tri-reagent (Molecular Research Centre Inc, USA) method using 100 mg of infected plant tissue [17,63,64]. PRSV-W positive isolates were also tested with WMV- and ZYMV-specific primers as described previously [13]. High-fidelity DNA polymerase enzyme (*Pfu*, Stratagene) was used along with Taq polymerase, and the number of PCR cycles was limited to 25 to reduce potential mutations generated during RT-PCR.

### 4.2. Cloning and Sequencing

The purified PCR products from each isolate were ligated in the pGEM-T Easy Vector (Promega Corp, Madison, WI, USA). The ligated products were transformed into *Escherichia coli* DH5α competent cells (New England Biolabs, Ipswich, MA, USA) and were subjected to blue–white screening using Luria-Bertani agar (LBA), carbenicillin, isopropyl-thiogalactopyranoside (IPTG), and X-gal. Three to five clones from each isolate were used for Sanger sequencing in both directions using applied biosystems 3130 at the Department of Biological Science, the University of Tulsa, Oklahoma [65]. The details of virus isolates, their host, geographical location, and collection year are depicted in Supplementary Table S1.

### 4.3. Sequence Analysis

The purified PCR products were sequenced in both forward and reverse directions using Sanger sequencing method. Sequences were retrieved, analyzed in Finch TV, and compared with GenBank sequences using the basic local alignment search tool (BLAST). Sequence alignment was completed for the clones using the Clustal X program and MEGAlign™ incorporated within the DNASTAR suite of programs (Madison, WI, USA). For CP sequences, consensus nt sequences for each isolate were obtained using the Editseq™. The consensus sequences of each virus isolate were deposited in the NCBI database, and their GenBank accession numbers are listed in Supplementary Table S1.

### 4.4. Phylogenetic Analysis

Consensus sequences of each isolate for CP gene of PRSV-W were used for phylogenetic analysis. The MEGA7 [66] software was used to construct four sets of ML phylogenetic trees: first, comparing PRSV-W isolates within this study (n = 101); second, comparing PRSV-W isolates from Oklahoma (101 isolates from this study and 64 selected isolates from GenBank); third, PRSV-W isolates from around the world (33 selected isolates from this study and 73 isolates from GenBank); and fourth, comparing all PRSV isolates irrespective of W or P strains (33 selected isolates from this study and 115 isolates from GenBank). The CP gene sequences of PRSV-W and PRSV-P retrieved from GenBank for phylogenetic analysis are listed in Supplementary Table S2. The best fit model selection test was completed in MEGA7, and the model with the lowest BIC value was selected for each phylogenetic tree reconstruction. For evaluation of statistical confidence in tree nodes, 1000 bootstrapping was completed. Each of these trees was visualized in Figtree version 1.4.3.

### 4.5. Genetic Diversity and Population Genetics

#### 4.5.1. Genetic Diversity

Genetic diversity of all CP sequences of PRSV-W isolates was determined based on host, geographical location, year of collection, and their respective phylogroups within Oklahoma using the Kimura 2 parameter in MEGA7. For each of the aforementioned categories, the number of haplotypes, haplotype diversity, number of segregating sites, average number of nt differences, and average nt diversity was calculated using DNASP6. These analyses were also completed separately for PRSV-W isolates and PRSV isolates (including both strains) from around the world.

#### 4.5.2. Gene Flow and Population Differentiation

The extents of genetic flow and differentiation were estimated using the fixation index (*Fst*) and the number of migrants successfully incoming per generation (Nm) values. The *Fst* value ranges from 0, indicating no genetic differentiation, to 1, indicating clear differentiation. The absolute *Fst* value of >0.33 indicates infrequent gene flow between the populations. Nm < 1 indicates reduced gene flow and increased genetic drift, which results in local population differentiation [67]. 

Similarly, the population differentiation was analyzed using permutation-based statistical tests Ks, Ks *, Z *, and Snn [67]. These tests are considered the most powerful statistical tests for analyzing sequence-based genetic differentiation among the highly mutating population in small sample sizes [68]. The Ks * was calculated as the average number of differences between sequences regardless of geographical origin. Under the null hypothesis, Kst * is expected to be near zero, meaning there is no genetic differentiation. The Z * statistic is a logarithmic variant of the rank statistic (Z), and smaller values indicate less genetic differentiation, and higher values with significant P values indicate higher genetic differentiation. The nearest neighbor statistic (Snn) measures the frequency of nearest neighbor sequences in the same locality. The value of Snn ranges from ½ in the case of panmixia to 1 when populations are distinctly differentiated. 

#### 4.5.3. Neutrality Tests

The values of segregating sites (S), the average number of nt differences (K), and a total number of mutations were used for testing the neutrality hypothesis using Tajima’s, Fu, and Li’s DandF among different population groups. Tajima’s D test is based on the differences between the two estimators, Tajima’s estimator (based on K) and Watterson’s estimator (based on S) [68]. The positive value on Tajima’s D statistic means an abundance of polymorphic alleles, while negative values indicate the presence of rare alleles. Fu and Li’s D test is based on the difference between singleton mutation sites and total mutation sites, and Fu and Li’s F test is based on a difference in singleton mutation sites and K [69]. The negative values for these tests mean a low-frequency polymorphism [70]. 

#### 4.5.4. Selection Analysis

Hyphy and Datamonkey packages were used for the isolate-selection analysis of different PRSV and PRSV-W populations. The dN/dS value was determined by the HyPhy packages included in MEGA7. The dN/dS value <1 shows evidence of the purifying selection, dN/dS=1 indicates neutral selection, and dN/dS >1 indicates positive selection. Similarly, FUBAR, FEL, MEME, and SLAC programs incorporated in Datamonkey (https://www.datamonkey.org, accessed on 5 March 2021 were used to deduce the numbers of positively and negatively selected codons. 

#### 4.5.5. Mutation Frequency and Pattern within the CP Gene

The total mutation count in the CP gene was completed for each isolate. The consensus nt sequence was inferred from 3–5 clones/isolate, and all mutations (substitution or indels) observed in those clones were counted. The total number of mutations within the different populations was determined, and mutation frequency was calculated using the following formula:

Mutation frequency = total number of mutations in n isolates with m clones/total number of nt in n × m

Whenever multiple nt mutations occurred consecutively, each individually mutated base was counted [43,71]. Any mutations that were only observed in either the forward or reverse direction were not considered as true mutations to reduce the possibility of artifact mutations. Mutation frequencies were determined for each isolate, location (county of collection), host, year of collection, and type of infection (single or mixed). For each of these categories, synonymous and non-synonymous mutations were also determined.

## Figures and Tables

**Table 1:** Nucleotide and amino acid identities among PRSV-W isolates obtained in this study and their comparison to PRSV isolates from other countries.

Geographical Location of Isolates	Number of Isolates Used for Analysis	Nt Identity (%)	Amino Acid Identity (%)
This study	101	96–100	98–100
Oklahoma	165 ^a^	96–100	98–100
Other parts of the USA	4	94–97	96–98
Australia	5	95–98	96–98
Bangladesh	2	88–90	87–93
Brazil	6	93–95	96–98
China	12	89–92	92–97
Columbia	2	93–95	95–96
Cuba	3	93–97	94–97
East Timor	3	91–92	95–96
France	1	95–97	97–98
India	16	86–95	89–97
Japan	5	89–91	91–94
Mexico	2	95–98	94–97
Myanmar	4	88–91	89–96
PNG	6	96–98	97–98
Poland	1	96–97	97–98
Taiwan	14	89–92	94–95
Thailand	2	90–91	92–93
South Korea	2	91–92	95–97
Venezuela	4	95–97	96–98

^a^ Represents isolates from this study and 64 isolates from the previous study [12].

**Table 2:** Population genetics analysis based on the coat protein gene sequences of PRSV-W isolates collected in different counties of Oklahoma: hosts, years of collection, and phylogroups.

County	No. of Sequences (n)	Mean Genetic Distance (d)	Haplotype Diversity (Hd)	Average # of Nt Difference (K)	Nt Diversity (π)	dN/dS
Blaine	99	0.023 ± 0.003	0.96	18.02	0.022	0.30
Caddo	49	0.001 ± 0.000	0.85	2.07	0.002	0.65
McCurtain	41	0.009 ± 0.002	0.87	10.97	0.013	0.28
Muskogee	112	0.016 ± 0.003	0.97	3.79	0.005	0.25
Cimarron	30	0.004 ± 0.001	0.95	7.14	0.009	0.95
Tulsa	39	0.006 ± 0.001	0.91	4.68	0.006	0.26
**Host**						
Cantaloupe	13	0.023 ± 0.003	0.96	16.59	0.020	0.17
Cucumber	10	0.021 ± 0.004	0.93	12.82	0.015	0.15
Pumpkin	217	0.016 ± 0.002	0.97	13.69	0.016	0.41
Squash	60	0.022 ± 0.003	0.96	16.83	0.020	0.25
Watermelon	72	0.020 ± 0.003	0.97	14.95	0.018	0.33
**Year of collection**						
2016	108	0.012 ± 0.002	0.93	10.56	0.013	0.47
2017	86	0.013 ± 0.002	0.96	11.69	0.014	0.37
2018	176	0.024 ± 0.003	0.98	18.21	0.022	0.36
**Phylogroups**						
Phylogroup 1	205	0.003 ± 0.001	0.96	3.47	0.004	0.72
Phylogroup 2	87	0.015 ± 0.003	0.97	12.90	0.015	0.44
Phylogroup 3	78	0.019 ± 0.004	0.92	13.04	0.016	0.35
**Overall**	**370**	**0.020 ± 0.003**	**0.98**	**16.09**	**0.019**	**0.36**

**Table 3:** Population genetic analysis based on the coat protein gene sequences of PRSV-W and PRSV populations in different phylogroups around the world.

Group	No. of Sequences (n)	Mean Genetic Distance (d)	Haplotype Diversity (Hd)	Average # of Nt Difference (K)	Nt Diversity (π)
**PRSV-W**					
This study	101	0.020 ± 0.003	0.95	16.12	0.02
Oklahoma ^a^	165	0.029 ± 0.004	0.95	16.57	0.02
Oceania	11	0.021 ± 0.003	0.96	17.91	0.02
Americas ^b^	11	0.051 ± 0.007	0.95	45.51	0.05
Asia	29	0.112 ± 0.014	0.99	79.86	0.09
**PRSV-W and P**					
Americas ^b^	22	0.060 ± 0.005	0.99	46.94	0.06
Asia	60	0.106 ± 0.008	0.99	74.95	0.09
PRSV-W ^c^	106	0.060 ± 0.005	0.99	51.71	0.07
PRSV-P	42	0.011 ± 0.008	0.99	75.47	0.09
**Overall ^d^**	148	0.085 ± 0.006	0.99	61.90	0.08

^a^ The sequences include 101PRSV-W isolates from this study and 64 PRSV-W isolates from GenBank. ^b^ The sequences exclude PRSV-W sequences from Oklahoma and include other isolates from North and South America. ^c^ Includes selected 33 PRSV-W consensus sequences from this study (out of 101) and 73 PRSV-W sequences retrieved from GenBank. ^d^ Includes selected 33 PRSV-W consensus sequences from this study (out of 101), 73 PRSV-W, and 42 PRSV-P sequences retrieved from GenBank.

**Table 4:** Gene flow and genetic differentiation estimates based on the coat protein gene sequences of PRSV-W isolates from different counties, hosts, phylogroups, and collection years.

Counties	*Fst*	Nm
BL vs. CD	**0.41**	0.36
BL vs. CM	**0.39**	0.39
BL vs. MC	0.24	0.78
BL vs. MK	**0.37**	0.42
BL vs. TL	**0.56**	0.20
CD vs. CM	**0.76**	0.08
CD vs. MC	**0.50**	0.25
CD vs. MK	0.14	1.60
CD vs. TL	**0.87**	0.04
CM vs. MC	**0.61**	0.16
CM vs. MK	**0.72**	0.10
CM vs. TL	**0.73**	0.09
MC vs. MK	**0.46**	0.29
MC vs. TL	**0.71**	0.10
MK vs. TL	**0.83**	0.05
Hosts		
CT vs. CU	0.15	1.42
CT vs. PM	0.17	1.24
CT vs. SQ	0.02	14.31
CT vs. WM	0.12	1.76
CU vs. PM	0.23	0.86
CU vs. SQ	0.11	2.02
CU vs. WM	0.25	0.74
PM vs. SQ	0.22	0.89
PM vs. WM	0.06	4.05
SQ vs. WM	0.18	1.14
Phylogroups		
PG1 vs. PG2	**0.64**	0.14
PG1 vs. PG3	**0.61**	0.16
PG2 vs. PG3	**0.54**	0.21
Collection years		
16 vs. 17	0.13	1.73
16 vs. 18	0.18	1.10
17 vs. 18	0.13	1.61

**Table 5:** Gene flow and genetic differentiation estimates based on the coat protein gene sequences of PRSV populations between different phylogroups from around the world.

Region/Phylogroups	*Fst*	Nm
PRSV-W populations		
Oklahoma vs. Americas	0.25	0.74
Oklahoma vs. Oceania	0.23	0.84
Oklahoma vs. Asia	0.43	0.34
Americas vs. Oceania	0.25	0.74
Americas vs. Asia	0.34	0.50
Oceania vs. Asia	0.44	0.32
PRSV-W and P populations		
Americas vs. Oceania	0.20	1.00
Americas vs. Asia	0.29	0.62
PRSV-P vs. PRSV-W	0.12	1.89

**Table 6:** Mutational statistics in the coat protein gene sequences of PRSV-W isolates from different counties of Oklahoma in single and mixed infections.

	Counties																	
	Blaine			Caddo			Cimarron			McCurtain			Muskogee			Tulsa		
	S ^a^	M ^b^	T ^c^	S	M	T	S	M	T	S	M	T	S	M	T	S	M	T
**No. of isolates**	10	16	26	1	12	13	1	8	9	7	5	12	26	5	31	1	9	10
**No. of clones**	39	60	99	4	45	49	4	26	30	23	18	41	94	18	112	5	34	39
**Mutation frequency (10^−3^)**	1.25	1.29	1.27	0.58	1.23	1.18	1.49	1.07	1.12	1.0	0.5	0.79	1.26	0.84	1.99	2.16	1.33	1.37
**dN/dS**	0.21	0.25	0.30	0.00	0.65	0.65	0.32	0.92	0.28	0.19	0.14	0.25	0.81	0.47	0.65	0.09	0.29	0.26

^a^ S, sequences originated from plants with single infection (PRSV-W only); ^b^ M, sequences originating from plants with mixed infection (PRSV-W together with WMV, ZYMV, or both); ^c^ T, all sequences, regardless of single or mixed infection.

**Table 7:** Mutational statistics in the coat protein gene sequences of PRSV-W isolates from different hosts in single and mixed infections.

	Host														
	Cantaloupe			Cucumber			Pumpkin			Squash			Watermelon		
	S	M	T	S	M	T	S	M	T	S	M	T	S	M	T
**No. of samples**	2	2	4	1	2	3	30	29	59	-	16	16	13	6	19
**No. of clones**	6	7	13	4	6	10	109	106	215	-	60	60	51	21	72
**Mutation frequency (10^−3^)**	1.16	1.16	1.16	1.45	1.54	1.5	1.25	1.24	1.24	-	0.98	0.98	1.13	1.05	1.11
**dN/dS**	0.45	0.16	0.17	0.32	0.95	0.11	0.33	0.35	0.41	-	0.25	0.25	0.34	0.17	0.33

S, sequences originated from plants with single infection (PRSV-W only); M, sequences originating from plants with mixed infection (PRSV-W together with WMV, ZYMV, or both); T, all sequences regardless of single or mixed infection.

**Table 8:** Mutational statistics in the coat protein gene sequences of PRSV-W clones from isolates collected in different years in single and mixed infections.

	Year											
	2016			2017			2018			Total		
	S ^a^	M ^b^	T ^c^	S	M	T	S	M	T	S	M	T
**No. of isolates**	14	15	29	16	9	25	16	31	47	46	55	101
**No. of clones**	52	56	108	56	30	86	61	115	176	169	201	370
**Mutation frequency (10^−3^)**	1.09	1.36	1.23	1.34	1.35	1.35	1.21	0.99	1.07	1.22	1.15	1.18
**dN/dS**	0.48	0.35	0.47	0.39	0.21	0.37	0.27	0.31	0.36	0.41	0.38	0.36

^a^ S, sequences originated from plants with single infection (PRSV-W only); ^b^ M, sequences originating from plants with mixed infection (PRSV-W together with WMV, ZYMV, or both); ^c^ T, all sequences regardless of single or mixed infection.

**Table 9:** Selection pressure analysis in the coat protein gene sequences among different PRSV populations.

PRSV Populations	Number of Sequences Used	Number of Negatively Selected Codons				Number of Positively Selected Codons			
		FUBAR	FEL	MEME	SLAC	FUBAR	FEL	MEME	SLAC
PRSV-W this study	101	12	27	-	4	2	0	0	0
PRSV-W Oklahoma	165 ^a^	28	50	-	13	2	0	0	0
PRSV-W global sequences	106 ^b^	214	214	-	197	3	3	11	3
PRSV global sequences	148 ^c^	229	250	-	205	3	3	13	5

^a^ Includes 101 PRSV-W sequences from this study and 64 PRSV-W sequences from GenBank. ^b^ Includes 33 PRSV-W sequences from this study, 73 PRSV-W sequences from GenBank. ^c^ Includes 33 PRSV-W sequences from this study, 73 PRSV-W, and 42 PRSV-P sequences from GenBank.

**Table 10:** List of the most frequent amino acid changes among 218 non-silent mutations observed in the coat protein gene sequences of PRSV-W isolates in this study.

Rank	Amino Acid Change	Frequency (%)
1	Lysine–Arginine	14 (6.34)
2	Asparagine–Aspartic acid	12 (5.43)
3	Arginine–Lysine	9 (4.07)
4	Alanine–Valine	8 (3.62)
4	Glutamic acid–Glycine	8 (3.62)
4	Leucine–Proline	8 (3.62)
7	Aspartic acid–Asparagine	7 (3.16)
7	Asparagine–Serine	7 (3.16)

**Table 11:** Mutational statistics of different regions of coat protein gene sequences of PRSV-W isolates.

	N-Terminal	Core	C-Terminal
Total nt sites	195	459	210
Mutation frequency	1.60 × 10^−3^	1.01 × 10^−3^	1.10 × 10^−3^
dN/dS	0.36	0.23	0.35
Conserved sites (%)	122 (62.6)	357 (77.8)	154 (13.3)
Silent sites (%)	26 (13.3)	39 (8.5)	21 (10)
Non-silent sites (%)	47 (24.1)	63 (13.7)	35 (16.7)
Mean genetic diversity (d)	0.019 ± 0.005	0.006 ± 0.001	0.009 ± 0.003

**Table 12:** Conserved amino acid motifs observed in PRSV population and their respective amino acid sequences with their corresponding positions within coat protein gene.

CP Region	Conserved Motif	Oklahoma	PRSV-W	PRSV	References
N-ter	DAG	7-**D[A/T]G**-9	7-**D[A/T/S]G**-9	7**-[D/N] [A/T/S]G**-9	[46]
N-ter	DVN[A/V]GT	61-**DVN[A/V]GT**-66	61-**DVN[A/V]GT**-66	61-**DVN[A/V]GT**-66	This study
Core	DISNTRAT	105-QI**DISNTRAT**QSQFEKWYEGV-125	107-**DISNTRAT**-114	107-**DISNTRAT**-114	This study
Core	MVWCI[E/D]NGTSP	129-DYGLNDNEMQVMLNGL**MVWCIENGTSP**DI-156	139-MLNGL**M[V/G]WCIENGTSP**D-155	139-MLNGL**M[V/G]WCIENGTSP**D-155	[56]
Core	W[V/T]MMDG[D/E/N]	157-SGV**WVMMDGE**-166	157-SGV**WVMM[D/G/E][G/E]E**-166	157-SGV**WVMM[D/G/E][G/E]E**-166	[57]
Core	FRQIMAHFSNAAEA	181-ATPS**FRQIMAHFSNAAEA**-198	185-**FRQIMAHFSNAAEA**-198	185-**FRQIMAHFSNAAEA**-198	This study
Core	[P/R/A]YMPRYG	208-**[R/G]YMPRYG**IKR-217	208-[**R/K/G]YMPRYG**[I/L]KR-217	208-**[R/K/G]YMPRYG**[I/L]KR-217	[58]
Core	YAFDFYE	219-LTDISLAR**[Y/H]AFDFYE**VNSKTP-239	221-DISLA**R[Y/H]AFDFYE**[V/I]NSKTP-239	221-D[I/T]SLAB[V/I]NSKTP-239	[59]
C-ter	QMKAAAL	246-HMQ**[M/V]KAAAL**R-255	248-**Q[M/V]KAAAL**R-255	248-**Q[M/V]KAAAL**R-255	[60,61,62]
C-ter	E[N/D]TERH	269-SNKEE**[N/D/S]TERH**TVEDVNR-280	272-E**E[N/D/S]TERH**TV-280	272-E**E[N/D/S]TERH**TV-280	[56,59]

## Data Availability

All 101 coat protein gene sequences of PRSV-W isolates presented in this study (Supplementary Table S1) were submitted to NCBI database. The accession numbers from (MZ099456-MZ099556) can be found online https://www.ncbi.nlm.nih.gov/genbank/ (accessed on 1 September 2021).

## Supplementary Materials

The following are available online at https://www.mdpi.com/article/10.3390/pathogens10101278/s1, Figure S1: Geographical locations of different counties of Oklahoma; Figure S2: Maximum likelihood (ML) phylogenetic tree constructed in MEGA 7 using general time reversible (GTR) model for coat protein gene of 165 PRSV-W isolates from Oklahoma (101 from this study and 64 from GenBank; Figure S3: Maximum likelihood (ML) phylogenetic tree constructed in MEGA 7 using general time reversible (GTR) model for coat protein gene of 148 PRSV isolates (33 from this study and 115 from GenBank). Table S1: List of papaya ringspot virus-W isolates collected and sequenced the coat protein gene in this study; Table S2: Nucleotide sequences of the coat protein gene of papaya ringspot virus isolates (W and P) retrieved from GenBank. Table S3: Estimates of evolutionary divergence among the coat protein gene sequences of papaya ringspot virus-W isolates collected from different counties of Oklahoma, hosts, collection years and phylogroups; Table S4: Estimates of evolutionary divergence among the coat protein gene sequences of papaya ringspot virus isolates (both W and P strains) different phylogroups; Table S5: Genetic differentiation estimates in the coat protein gene sequences of papaya ringspot virus-W isolates from different counties, hosts, phylogroups and collection years; Table S6: Neutrality tests of coat protein gene sequences among the PRSV-W isolates from different counties, hosts, phylogroups and collection years; Table S7: Gene flow and genetic differentiation estimates based on the coat protein gene sequences of papaya ringspot virus –W isolates between different phylogroups from around the world; Table S8: Neutrality tests of coat protein gene sequences of papaya ringspot virus isolates among different population groups.
