# Supplementary material for: High Mutation Frequency and Significant Population Differentiation in Papaya Ringspot Virus-W Isolates
Source: Pathogens. 2021 Oct 4;10(10):1278. doi: 10.3390/pathogens10101278 (PMC8537659; doi:10.3390/pathogens10101278)
Supplement: Supplementary file 1 [file pathogens-10-01278-s001.zip › pathogens-1385613-supplementary.pdf]

**Table S1**: List of papaya ringspot virus-W isolates collected and sequenced the coat protein gene in this study

| No. | Isolate name | County | Year of collection | Host | Single[a]/mixed[b] infection | GenBank Accession number |
|---|---|---|---|---|---|---|
| 1 | BL17 | Blaine | 2016 | Pumpkin | Mixed | MZ099456 |
| 2 | BL18 | Blaine | 2016 | Pumpkin | Mixed | MZ099457 |
| 3 | BL19 | Blaine | 2016 | Pumpkin | Mixed | MZ099458 |
| 4 | BL20 | Blaine | 2016 | Pumpkin | Single | MZ099459 |
| 5 | BL21 | Blaine | 2016 | Pumpkin | Single | MZ099460 |
| 6 | BL22 | Blaine | 2016 | Pumpkin | Single | MZ099461 |
| 7 | BL23 | Blaine | 2016 | Pumpkin | Single | MZ099462 |
| 8 | BL24 | Blaine | 2016 | Pumpkin | Mixed | MZ099463 |
| 9 | BL25 | Blaine | 2017 | Pumpkin | Mixed | MZ099464 |
| 10 | BL26 | Blaine | 2017 | Pumpkin | Mixed | MZ099465 |
| 11 | BL27 | Blaine | 2017 | Pumpkin | Single | MZ099466 |
| 12 | BL28 | Blaine | 2017 | Pumpkin | Single | MZ099467 |
| 13 | BL29 | Blaine | 2017 | Pumpkin | Mixed | MZ099468 |
| 14 | BL30 | Blaine | 2017 | Pumpkin | Mixed | MZ099469 |
| 15 | BL31 | Blaine | 2017 | Pumpkin | Mixed | MZ099470 |
| 16 | BL32 | Blaine | 2017 | Pumpkin | Single | MZ099471 |
| 17 | BL33 | Blaine | 2017 | Pumpkin | Mixed | MZ099472 |
| 18 | BL34 | Blaine | 2017 | Pumpkin | Single | MZ099473 |
| 19 | BL35 | Blaine | 2018 | Pumpkin | Mixed | MZ099474 |
| 20 | BL36 | Blaine | 2018 | Watermelon | Single | MZ099475 |
| 21 | BL37 | Blaine | 2018 | Pumpkin | Mixed | MZ099476 |
| 22 | BL38 | Blaine | 2018 | Pumpkin | Single | MZ099477 |
| 23 | BL39 | Blaine | 2018 | Pumpkin | Mixed | MZ099478 |
| 24 | BL40 | Blaine | 2018 | Pumpkin | Mixed | MZ099479 |
| 25 | BL41 | Blaine | 2018 | Pumpkin | Mixed | MZ099480 |
| 26 | BL42 | Blaine | 2018 | Pumpkin | Mixed | MZ099481 |
| 27 | CD1 | Caddo | 2016 | Pumpkin | Mixed | MZ099482 |
| 28 | CD2 | Caddo | 2016 | Pumpkin | Mixed | MZ099483 |
| 29 | CD3 | Caddo | 2016 | Pumpkin | Mixed | MZ099484 |
| 30 | CD4 | Caddo | 2016 | Pumpkin | Mixed | MZ099485 |
| 31 | CD5 | Caddo | 2016 | Pumpkin | Mixed | MZ099486 |
| 32 | CD6 | Caddo | 2016 | Squash | Mixed | MZ099487 |
| 33 | CD7 | Caddo | 2016 | Squash | Mixed | MZ099488 |
| 34 | CD8 | Caddo | 2016 | Squash | Mixed | MZ099489 |
| 35 | CD9 | Caddo | 2016 | Watermelon | Mixed | MZ099490 |
| 36 | CD10 | Caddo | 2017 | Pumpkin | Mixed | MZ099491 |
| 37 | CD11 | Caddo | 2017 | Pumpkin | Single | MZ099492 |
| 38 | CD12 | Caddo | 2017 | Pumpkin | Mixed | MZ099493 |
| 39 | CD13 | Caddo | 2017 | Pumpkin | Mixed | MZ099494 |
| 40 | CM1 | Cimarron | 2018 | Cucumber | Single | MZ099495 |
| 41 | CM2 | Cimarron | 2018 | Squash | Mixed | MZ099496 |
| 42 | CM3 | Cimarron | 2018 | Cucumber | Mixed | MZ099497 |
| 43 | CM4 | Cimarron | 2018 | Cucumber | Mixed | MZ099498 |
| 44 | CM5 | Cimarron | 2018 | Squash | Mixed | MZ099499 |
| 45 | CM6 | Cimarron | 2018 | Squash | Mixed | MZ099500 |
| 46 | CM7 | Cimarron | 2018 | Squash | Mixed | MZ099501 |
| 47 | CM8 | Cimarron | 2018 | Squash | Mixed | MZ099502 |
| 48 | CM9 | Cimarron | 2018 | Squash | Mixed | MZ099503 |

| | | | | | | |
|---|---|---|---|---|---|---|
| 49 | MC1 | McCurtain | 2016 | Watermelon | Mixed | MZ099504 |
| 50 | MC2 | McCurtain | 2016 | Watermelon | Single | MZ099505 |
| 51 | MC3 | McCurtain | 2016 | Pumpkin | Mixed | MZ099506 |
| 52 | MC4 | McCurtain | 2018 | Watermelon | Single | MZ099507 |
| 53 | MC5 | McCurtain | 2018 | Watermelon | Single | MZ099508 |
| 54 | MC6 | McCurtain | 2018 | Watermelon | Single | MZ099509 |
| 55 | MC7 | McCurtain | 2018 | Squash | Mixed | MZ099510 |
| 56 | MC8 | McCurtain | 2018 | Pumpkin | Single | MZ099511 |
| 57 | MC9 | McCurtain | 2018 | Watermelon | Mixed | MZ099512 |
| 58 | MC10 | McCurtain | 2018 | Watermelon | Mixed | MZ099513 |
| 59 | MC11 | McCurtain | 2018 | Pumpkin | Single | MZ099514 |
| 60 | MC12 | McCurtain | 2018 | Cantaloupe | Single | MZ099515 |
| 61 | MK1 | Muskogee | 2016 | Watermelon | Single | MZ099516 |
| 62 | MK2 | Muskogee | 2016 | Pumpkin | Single | MZ099517 |
| 63 | MK3 | Muskogee | 2016 | Pumpkin | Single | MZ099518 |
| 64 | MK4 | Muskogee | 2016 | Watermelon | Single | MZ099519 |
| 65 | MK5 | Muskogee | 2016 | Pumpkin | Single | MZ099520 |
| 66 | MK6 | Muskogee | 2016 | Pumpkin | Single | MZ099521 |
| 67 | MK7 | Muskogee | 2016 | Pumpkin | Single | MZ099522 |
| 68 | MK8 | Muskogee | 2016 | Pumpkin | Single | MZ099523 |
| 69 | MK9 | Muskogee | 2016 | Pumpkin | Single | MZ099524 |
| 70 | MK10 | Muskogee | 2017 | Pumpkin | Single | MZ099525 |
| 71 | MK11 | Muskogee | 2017 | Pumpkin | Single | MZ099526 |
| 72 | MK12 | Muskogee | 2017 | Pumpkin | Single | MZ099527 |
| 73 | MK13 | Muskogee | 2017 | Pumpkin | Single | MZ099528 |
| 74 | MK14 | Muskogee | 2017 | Pumpkin | Single | MZ099529 |
| 75 | MK15 | Muskogee | 2017 | Pumpkin | Single | MZ099530 |
| 76 | MK16 | Muskogee | 2017 | Pumpkin | Single | MZ099531 |
| 77 | MK17 | Muskogee | 2017 | Pumpkin | Single | MZ099532 |
| 78 | MK18 | Muskogee | 2017 | Pumpkin | Single | MZ099533 |
| 79 | MK19 | Muskogee | 2017 | Pumpkin | Single | MZ099534 |
| 80 | MK20 | Muskogee | 2017 | Pumpkin | Single | MZ099535 |
| 81 | MK21 | Muskogee | 2018 | Cantaloupe | Single | MZ099536 |
| 82 | MK22 | Muskogee | 2018 | Watermelon | Single | MZ099537 |
| 83 | MK23 | Muskogee | 2018 | Watermelon | Single | MZ099538 |
| 84 | MK24 | Muskogee | 2018 | Watermelon | Single | MZ099539 |
| 85 | MK24 | Muskogee | 2018 | Watermelon | Single | MZ099540 |
| 86 | MK26 | Muskogee | 2018 | Watermelon | Single | MZ099541 |
| 87 | MK27 | Muskogee | 2018 | Squash | Mixed | MZ099542 |
| 88 | MK28 | Muskogee | 2018 | Pumpkin | Mixed | MZ099543 |
| 89 | MK29 | Muskogee | 2018 | Pumpkin | Mixed | MZ099544 |
| 90 | MK30 | Muskogee | 2018 | Pumpkin | Mixed | MZ099545 |
| 91 | MK31 | Muskogee | 2018 | Pumpkin | Mixed | MZ099546 |
| 92 | TL17 | Tulsa | 2018 | Squash | Mixed | MZ099547 |
| 93 | TL18 | Tulsa | 2018 | Cantaloupe | Mixed | MZ099548 |
| 94 | TL19 | Tulsa | 2018 | Squash | Mixed | MZ099549 |
| 95 | TL20 | Tulsa | 2018 | Squash | Mixed | MZ099550 |
| 96 | TL21 | Tulsa | 2018 | Squash | Mixed | MZ099551 |
| 97 | TL22 | Tulsa | 2018 | Squash | Mixed | MZ099552 |
| 98 | TL23 | Tulsa | 2018 | Watermelon | Single | MZ099553 |
| 99 | TL24 | Tulsa | 2018 | Watermelon | Mixed | MZ099554 |
| 100 | TL25 | Tulsa | 2018 | Watermelon | Mixed | MZ099555 |
| 101 | TL26 | Tulsa | 2018 | Cantaloupe | Mixed | MZ099556 |

a= Infection with PRSV-W only, b=infection with PRSV-W in addition to WMV/ZYMV or both

**Table S2**: Nucleotide sequences of the coat protein gene of papaya ringspot virus isolates (W and P) retrieved from GenBank

| # | Isolate | Country | Host | GenBank Accession |
|---|---|---|---|---|
| 1 | - | Australia | Pumpkin | S89893 |
| 2 | 23C | Australia | Watermelon | KX655861 |
| 3 | 38NT | Australia | Honeydew | KX655872 |
| 4 | 51C | Australia | Pumpkin | KX655862 |
| 5 | B9 | Australia | Pumpkin | KX655867 |
| 6 | BD2 | Bangladesh | Papaya | MH397222 |
| 7 | BD1 | Bangladesh | Papaya | MH444652 |
| 8 | PRSV-W-2 | Brazil | Zucchini squash | AF530088 |
| 9 | PRSV-W-2R | Brazil | Zucchini squash | AF530089 |
| 10 | PRSV-W-1 | Brazil | Zucchini squash | AY094987 |
| 11 | FEV | Brazil | Fevillea | KP462721 |
| 12 | -(WM) | Brazil | Watermelon | MN364666 |
| 13 | -(Mel) | Brazil | Melon | MG030689 |
| 14 | BN | China | Pumpkin | DQ449533 |
| 15 | Ch99/113 | China | Squash | DQ868880 |
| 16 | BS10 | China | Pumpkin | MK988418 |
| 17 | Hn | China | Pumpkin | MF074214 |
| 18 | SD | China | Pumpkin | MF085000 |
| 19 | XM | China | Papaya | KY933061 |
| 20 | P | China | Papaya | EF183499 |
| 21 | Hainan | China | Papaya | KF734962 |
| 22 | LM | China | Papaya | KT633943 |
| 23 | HN1 | China | Papaya | HQ424465 |
| 24 | HNVb | China | Papaya | KF791028 |
| 25 | HNDF | China | Papaya | KT895257 |
| 26 | CH | Columbia | Papaya | KT275938 |
| 27 | VR | Columbia | Papaya | KT275937 |
| 28 | Mch | Cuba | Bitter melon | KP019380 |
| 29 | Boyeros-P | Cuba | Papaya | DQ089482 |
| 30 | Bejucal | Cuba | Papaya | MF041942 |
| 31 | TM4 | East Timor | Pumpkin | KX655864 |
| 32 | TM3 | East Timor | Pumpkin | KX655865 |
| 33 | TM50 | East Timor | Cucumber | KX655874 |
| 34 | E2 | France | Pumpkin | KC345609 |
| 35 | SG1 | India | Snake Gourd | KP161501 |
| 36 | Pum1 | India | Pumpkin | KP161500 |
| 37 | Tiru1 | India | Pumpkin | MH374912 |
| 38 | CuCu2 | India | Cucumber | KP161498 |
| 39 | Pum5 | India | Pumpkin | KP161497 |
| 40 | Meghalaya | India | Papaya | MF356497 |
| 41 | WB | India | Papaya | LC482263 |
| 42 | PMH | India | Papaya | MF405295 |
| 43 | PMI | India | Papaya | MF405296 |
| 44 | PS3H | India | Papaya | MF405297 |
| 45 | PS3I | India | Papaya | MF405298 |
| 46 | VC | India | Papaya | MF405299 |
| 47 | Pune | India | Papaya | MH311882 |
| 48 | R3 | India | Papaya | KJ755852 |
| 49 | HYD | India | Papaya | KP743981 |

| | | | | |
|---|---|---|---|---|
| 50 | DEL | India | Papaya | EF017707 |
| 51 | JTK9 | Japan | Trichosanthes | AB583216 |
| 52 | JIK2 | Japan | Trichosanthes | AB583217 |
| 53 | JIK5 | Japan | Trichosanthes | AB583218 |
| 54 | JTK7 | Japan | Trichosanthes | AB583219 |
| 55 | JTK8 | Japan | Trichosanthes | AB583220 |
| 56 | CDVA | Mexico | Papaya | MN203186 |
| | CDPS | Mexico | Papaya | MN203187 |
| 57 | VrPO | Mexico | Papaya | AY231130 |
| 58 | Mp18 | Myanmar | Pumpkin | AB583208 |
| 59 | Mcu11 | Myanmar | Cucumber | AB583209 |
| 60 | Mp6 | Myanmar | Pumpkin | AB583210 |
| 61 | Mbo2 | Myanmar | Bottle gourd | AB583211 |
| 62 | BON | Poland | Zucchini | GQ927328 |
| 63 | 8A | PNG | Pumpkin | MH4044259 |
| 64 | 12B | PNG | Pumpkin | MH4044260 |
| 65 | 16A | PNG | Pumpkin | MH4044261 |
| 66 | 17B | PNG | Cucumber | MH4044262 |
| 67 | 18B | PNG | Pumpkin | MH4044263 |
| 68 | 22A | PNG | Pumpkin | MH4044264 |
| 69 | KW | South Korea | Cucumber | AB369277 |
| 70 | SK | South Korea | Pumpkin | KY996464 |
| 71 | PT | Taiwan | Melon | AY027811 |
| 72 | TN | Taiwan | Sponge gourd | AY027812 |
| 73 | YK | Taiwan | - | X78557 |
| 74 | CI | Taiwan | Sponge Gourd | AY027810 |
| 75 | NPh | Taiwan | Papaya | JX448370 |
| 76 | Ad6 | Taiwan | Papaya | JX448369 |
| 77 | Leaf mottling | Taiwan | Papaya | DQ340770 |
| 78 | Leaf Deformation | Taiwan | Papaya | DQ340769 |
| 79 | MPh | Taiwan | Papaya | JX448371 |
| 80 | DPh | Taiwan | Papaya | JX448372 |
| 81 | NP | Taiwan | Papaya | JX448373 |
| 82 | Leaf Deformation2 | Taiwan | Papaya | DQ340771 |
| 83 | YK | Taiwan | Papaya | X97251 |
| 84 | P519 | Taiwan | Papaya | EU882728 |
| 85 | Thai | Thailand | - | AY010722 |
| 86 | P | Thailand | Papaya | AY162218 |
| 87 | W1 | USA | - | D00594 |
| 88 | HA | USA | Papaya | X67673, S46722 |
| 89 | PG | USA | Papaya | EU126128 |
| 90 | PTX | USA | Papaya | KY271954 |
| 91 | A1a | USA | Watermelon | JN132408 |
| 92 | A1b | USA | Watermelon | JN132409 |
| 93 | A2 | USA | Watermelon | JN132410 |
| 94 | A3 | USA | Watermelon | JN132411 |
| 95 | A4 | USA | Watermelon | JN132412 |
| 96 | A5 | USA | Watermelon | JN132413 |
| 97 | A6 | USA | Watermelon | JN132414 |
| 98 | A7 | USA | Watermelon | JN132415 |
| 99 | A8 | USA | Watermelon | JN132416 |
| 100 | A9 | USA | Watermelon | JN132417 |
| 101 | A10 | USA | Watermelon | JN132418 |

| | | | | |
|---|---|---|---|---|
| 102 | A11 | USA | Watermelon | JN132419 |
| 103 | A12 | USA | Watermelon | JN132420 |
| 104 | A13 | USA | Watermelon | JN132421 |
| 105 | A14 | USA | Watermelon | JN132422 |
| 106 | A15 | USA | Watermelon | JN132423 |
| 107 | B1 | USA | Watermelon | JN132424 |
| 108 | B2 | USA | Watermelon | JN132425 |
| 109 | B3 | USA | Watermelon | JN132426 |
| 110 | B4 | USA | Watermelon | JN132427 |
| 111 | B5 | USA | Watermelon | JN132428 |
| 112 | B6 | USA | Watermelon | JN132429 |
| 113 | B7 | USA | Watermelon | JN132430 |
| 114 | B8 | USA | Watermelon | JN132431 |
| 115 | B9 | USA | Watermelon | JN132432 |
| 116 | B10 | USA | Watermelon | JN132433 |
| 117 | B11 | USA | Watermelon | JN132434 |
| 118 | B12 | USA | Watermelon | JN132435 |
| 119 | B13 | USA | Watermelon | JN132436 |
| 120 | B14 | USA | Watermelon | JN132437 |
| 121 | B15 | USA | Watermelon | JN132438 |
| 122 | J1 | USA | Watermelon | JN132439 |
| 123 | J2 | USA | Watermelon | JN132440 |
| 124 | J3 | USA | Watermelon | JN132441 |
| 125 | J4 | USA | Watermelon | JN132442 |
| 126 | J5 | USA | Watermelon | JN132443 |
| 127 | J6 | USA | Watermelon | JN132444 |
| 128 | J7 | USA | Watermelon | JN132445 |
| 129 | J8 | USA | Watermelon | JN132446 |
| 130 | J9 | USA | Watermelon | JN132447 |
| 131 | J10 | USA | Watermelon | JN132449 |
| 132 | J11 | USA | Watermelon | JN132449 |
| 133 | J12 | USA | Watermelon | JN132450 |
| 134 | J13 | USA | Watermelon | JN132451 |
| 135 | J14 | USA | Watermelon | JN132452 |
| 136 | J15a | USA | Watermelon | JN132453 |
| 137 | J15b | USA | Watermelon | JN132454 |
| 138 | T1 | USA | Watermelon | JN132455 |
| 139 | T2 | USA | Watermelon | JN132456 |
| 140 | T3 | USA | Watermelon | JN132457 |
| 141 | T4 | USA | Watermelon | JN132458 |
| 142 | T5 | USA | Watermelon | JN132459 |
| 143 | T6 | USA | Watermelon | JN132460 |
| 144 | T7 | USA | Watermelon | JN132461 |
| 145 | T8 | USA | Watermelon | JN132462 |
| 146 | T9 | USA | Watermelon | JN132463 |
| 147 | T10 | USA | Watermelon | JN132464 |
| 148 | T11a | USA | Watermelon | JN132465 |
| 149 | T11b | USA | Watermelon | JN132466 |
| 150 | T12 | USA | Watermelon | JN132467 |
| 151 | T13 | USA | Watermelon | JN132468 |
| 152 | T14a | USA | Watermelon | JN132469 |
| 153 | T14b | USA | Watermelon | JN132470 |
| 154 | T15 | USA | Watermelon | JN132471 |

| | | | | |
|---|---|---|---|---|
| 155 | VE10-234 | Venezuela | Watermelon | KC345595 |
| 156 | VE10-207 | Venezuela | Watermelon | KC345594 |
| 157 | VE08-56 | Venezuela | Pumpkin | KC345585 |
| 158 | Merida-6 | Venezuela | Papaya | EF189736 |

**Table S3:** Estimates of evolutionary divergence among the coat protein gene sequences of papaya ringspot virus-W isolates collected from different counties of Oklahoma, hosts, collection years and phylogroups

| **Counties** | Caddo | Cimarron | McCurtain | Muskogee | Tulsa |
|---|---|---|---|---|---|
| Blaine | 0.020±0.003 | 0.026±0.004 | 0.025±0.004 | 0.021±0.003 | 0.032±0.005 |
| Caddo | | 0.024±0.005 | 0.017±0.003 | 0.003±0.001 | 0.029±0.005 |
| Cimarron | | | 0.031±0.006 | 0.024±0.005 | 0.026±0.005 |
| McCurtain | | | | 0.018±0.003 | 0.036±0.006 |
| Muskogee | | | | | 0.030±0.005 |
| | | | | | |
| **Hosts** | Cucumber | Pumpkin | Squash | Watermelon | |
| Cantaloupe | 0.021±0.004 | 0.021±0.003 | 0.021±0.003 | 0.022±0.003 | |
| Cucumber | | 0.023±0.004 | 0.021±0.003 | 0.026±0.004 | |
| Pumpkin | | | 0.024±0.003 | 0.019±0.002 | |
| Squash | | | | 0.025±0.003 | |
| | | | | | |
| **Year** | 2017 | 2018 | | | |
| 2016 | 0.014±0.002 | 0.022±0.003 | | | |
| 2017 | | 0.021±0.003 | | | |
| | | | | | |
| **Phylogroups** | Phylogroup 2 | Phylogroup 3 | | | |
| Phylogroup 1 | 0.027±0.004 | 0.024±0.004 | | | |
| Phylogroup 2 | | 0.036±0.005 | | | |

**Table S4:** Estimates of evolutionary divergence among the coat protein gene sequences of papaya ringspot virus isolates (both W and P strains) different phylogroups

| **PRSV-W group** | Americas W | Oceania W | Asia W |
|---|---|---|---|
| Oklahoma W | 0.047±0.006 | 0.028±0.004 | 0.115±0.015 |
| Americas W | | 0.046± 0.006 | 0.12± 0.016 |
| Oceania W | | | 0.117±0.016 |
| | | | |
| **PRSV group** | Americas | Oceania | Asia |
| Oklahoma | 0.050±0.004 | 0.028±0.004 | 0.110±0.009 |
| Americas | | 0.051±0.004 | 0.118±0.010 |
| Oceania | | | 0.11±0.010 |

**Table S5:** Genetic differentiation estimates in the coat protein gene sequences of papaya ringspot virus-W isolates from different counties, hosts, phylogroups and collection years

| Counties | Ks | Kst | P-value | Ks* | Kst* | P-Value | Z* | P value | Snn | P-value |
|---|---|---|---|---|---|---|---|---|---|---|
| BL vs CD | 12.74 | 0.20 | 0.000*** | 2.10 | 0.15 | 0.000*** | 7.96 | 0.000*** | 0.93 | 0.000*** |
| BL vs CM | 16.43 | 0.16 | 0.000*** | 2.46 | 0.10 | 0.000*** | 7.74 | 0.000*** | 1.00 | 0.000*** |
| BL vs MC | 17.08 | 0.11 | 0.000*** | 2.49 | 0.08 | 0.000*** | 7.96 | 0.000*** | 0.92 | 0.000*** |
| BL vs MK | 10.94 | 0.24 | 0.000*** | 2.04 | 0.15 | 0.000*** | 8.70 | 0.000*** | 0.95 | 0.000*** |
| BL vs TL | 15.39 | 0.29 | 0.000*** | 2.32 | 0.18 | 0.000*** | 7.63 | 0.000*** | 1.00 | 0.000*** |
| CD vs CM | 4.00 | 0.63 | 0.000*** | 1.25 | 0.38 | 0.000*** | 6.55 | 0.000*** | 0.96 | 0.000*** |
| CD vs MC | 6.35 | 0.35 | 0.000*** | 1.43 | 0.22 | 0.000*** | 7.04 | 0.000*** | 0.77 | 0.000*** |
| CD vs MK | 3.27 | 0.06 | 0.000*** | 1.33 | 0.04 | 0.000*** | 8.39 | 0.000*** | 0.76 | 0.000*** |
| CD vs TL | 3.23 | 0.77 | 0.000*** | 1.16 | 0.47 | 0.000*** | 6.62 | 0.000*** | 1.00 | 0.000*** |
| CM vs MC | 10.51 | 0.42 | 0.000*** | 1.89 | 0.26 | 0.000*** | 6.33 | 0.000*** | 0.93 | 0.000*** |
| CM vs MK | 4.62 | 0.51 | 0.000*** | 1.52 | 0.23 | 0.000*** | 7.91 | 0.000*** | 0.97 | 0.000*** |
| CM vs TL | 5.94 | 0.58 | 0.000*** | 1.51 | 0.34 | 0.000*** | 6.17 | 0.000*** | 1.00 | 0.000*** |
| MC vs MK | 6.04 | 0.31 | 0.000*** | 1.62 | 0.15 | 0.000*** | 8.16 | 0.000*** | 0.84 | 0.000*** |
| MC vs TL | 8.77 | 0.55 | 0.000*** | 1.71 | 0.34 | 0.000*** | 6.42 | 0.000*** | 1.00 | 0.000*** |
| MK vs TL | 4.05 | 0.67 | 0.000*** | 1.45 | 0.33 | 0.000*** | 7.83 | 0.000*** | 1.00 | 0.000*** |
| **Hosts** | | | | | | | | | | |
| CT vs CU | 15.43 | 0.08 | 0.046* | 2.41 | 0.06 | 0.018* | 4.42 | 0.025* | 0.78 | 0.017* |
| CT vs PM | 13.85 | 0.02 | 0.001** | 2.34 | 0.001 | 0.001** | 9.15 | 0.00*** | 0.97 | 0.000*** |
| CT vs SQ | 17.34 | 0.01 | 0.25ns | 2.56 | 0.01 | 0.079ns | 6.87 | 0.12ns | 0.81 | 0.004** |
| CT vs WM | 16.12 | 0.04 | 0.007** | 2.45 | 0.02 | 0.011* | 7.13 | 0.004** | 0.85 | 0.000*** |
| CU vs PM | 13.65 | 0.02 | 0.001** | 2.33 | 0.01 | 0.000*** | 9.13 | 0.000*** | 0.98 | 0.000*** |
| CU vs SQ | 17.05 | 0.03 | 0.006** | 2.54 | 0.02 | 0.028* | 6.74 | 0.008** | 0.92 | 0.000*** |
| CU vs WM | 15.87 | 0.07 | 0.000*** | 2.42 | O,o4 | 0.000*** | 7.04 | 0.000*** | 0.95 | 0.000*** |
| PM vs SQ | 14.37 | 0.09 | 0.000*** | 2.38 | 0.05 | 0.000*** | 9.43 | 0.000*** | 0.88 | 0.000*** |
| PM vs WM | 14.00 | 0.02 | 0.000*** | 2.34 | 0.02 | 0.000*** | 9.60 | 0.000*** | 0.83 | 0.000*** |
| SQ vs WM | 16.79 | 0.09 | 0.000*** | 2.50 | 0.06 | 0.000*** | 7.93 | 0.000*** | 0.73 | 0.000*** |
| **Phylogroups** | | | | | | | | | | |
| PG1 vs PG2 | 6.26 | 0.49 | 0.000*** | 1.65 | 0.26 | 0.000*** | 9.26 | 0.000*** | 1.00 | 0.000*** |
| PG1 vs PG3 | 6.09 | 0.46 | 0.000*** | 1.60 | 0.25 | 0.000*** | 9.20 | 0.000*** | 1.00 | 0.000*** |
| PG2 vs PG3 | 13.99 | 0.37 | 0.000*** | 2.27 | 0.21 | 0.000*** | 7.85 | 0.000*** | 1.00 | 0.000*** |
| **Collection years** | | | | | | | | | | |
| 16 vs 17 | 11.07 | 0.07 | 0.000*** | 2.04 | 0.04 | 0.000*** | 8.80 | 0.000*** | 0.78 | 0.000*** |
| 16 vs 18 | 15.28 | 0.09 | 0.000*** | 2.39 | 0.08 | 0.000*** | 9.39 | 0.000*** | 0.95 | 0.000*** |
| 17 vs 18 | 16.87 | 0.06 | 0.000*** | 2.55 | 0.04 | 0.000*** | 9.30 | 0.000*** | 0.95 | 0.000*** |

*p* value estimates are based on probability obtained by the permutation test with 1000 replicates. ns; not significant, *;0.01< $p$ <0.05, **; 0.001< $p$ <0.01, ***; $p$ <0.001. $p$ >0.001, significantly rejects the null hypothesis that there is no genetic differentiation between two populations

BL=Blaine, CD= Caddo, CM=Cimarron, MC= McCurtain, MK=Muskogee, TL=Tulsa

**Table S6:** Neutrality tests of coat protein gene sequences among the PRSV-W isolates from different counties, hosts, phylogroups and collection years

| **Counties** | Fu and Li's D | Fu and Li's F | Tajima's D |
|---|---|---|---|
| Blaine | -2.47* | -2.16 ns | -0.87 ns |
| Caddo | -4.51** | -4.52** | -2.48 ** |
| Cimarron | -0.35 ns | -0.75 ns | -1.23 ns |
| McCurtain | -0.97 ns | -0.52 ns | 0.46 ns |
| Muskogee | -4.03** | -4.04 ** | -2.48** |
| Tulsa | -0.64 ns | -1.28 ns | -1.92* |
| **Hosts** | | | |
| Cantaloupe | -5.51** | -4.24** | 1.28ns |
| Cucumber | 0.94ns | 1.16ns | 1.29ns |
| Pumpkin | -4.19** | -3.62** | -1.85* |
| Squash | -1.80ns | -1.51ns | -0.41ns |
| Watermelon | -1.68ns | -1.51ns | -0.63ns |
| **Phylogroups** | | | |
| Phylogroup 1 | -6.37** | -5.50** | -2.65** |
| Phylogroup 2 | -2.48* | -2.08ns | -0.66ns |
| Phylogroup 3 | -2.80ns | -1.74ns | -0.53ns |
| **Collection year** | | | |
| 2016 | -4.96** | -4.31** | -1.79* |
| 2017 | -2.44* | -2.44* | -1.84* |
| 2018 | -3.38** | -2.70** | -1.91* |
| Overall | -5.50** | -4.24** | -1.91* |

ns; not significant,*;0.02<P<0.05, **;P<0.02

**Table S7:** Gene flow and genetic differentiation estimates based on the coat protein gene sequences of papaya ringspot virus –W isolates between different phylogroups from around the world

| Region/phylogroups | Ks | Kst | P-value | Ks* | Kst* | P-Value | Z* | P value | Snn | P-value |
|---|---|---|---|---|---|---|---|---|---|---|
| Oklahoma vs Americas | 22.82 | 0.12 | 0.000*** | 2.91 | 0.05 | 0.000*** | 6.40 | 0.000*** | 0.95 | 0.000*** |
| Oklahoma vs Oceania | 18.79 | 0.08 | 0.000*** | 2.80 | 0.04 | 0.000*** | 6.43 | 0.000*** | 0.98 | 0.000*** |
| Oklahoma vs Asia | 40.06 | 0.29 | 0.000*** | 3.26 | 0.12 | 0.000*** | 6.72 | 0.000*** | 1.00 | 0.000*** |
| Americas vs Oceania | 30.58 | 0.15 | 0.000*** | 3.23 | 0.07 | 0.000*** | 4.03 | 0.000*** | 0.89 | 0.000*** |
| Americas vs Asia | 69.32 | 0.15 | 0.000*** | 4.04 | 0.05 | 0.000*** | 5.24 | 0.000*** | 1.00 | 0.000*** |
| Oceania vs Asia | 62.88 | 0.20 | 0.000*** | 3.85 | 0.08 | 0.000*** | 5.12 | 0.000*** | 1.00 | 0.000*** |
| Americas vs Oceania | 38.59 | 0.09 | 0.000*** | 3.52 | 0.04 | 0.000*** | 4.92 | 0.000*** | 0.95 | 0.000*** |
| Americas vs Asia | 67.43 | 0.13 | 0.000*** | 4.08 | 0.04 | 0.000*** | 6.75 | 0.000*** | 1.00 | 0.000*** |
| PRSV-P vs PRSV-W | 58.46 | 0.06 | 0.000*** | 3.83 | 0.03 | 0.000*** | 8.12 | 0.000*** | 0.92 | 0.000*** |

*p* value estimates are based on probability obtained by the permutation test with 1000 replicates. ns; not significant, *;0.01< $p$ <0.05, **; 0.001< $p$ <0.01, ***; $p$ <0.001. $p$ >0.001, significantly rejects the null hypothesis that there is no genetic differentiation between two populations

**Table S8:** Neutrality tests of coat protein gene sequences of papaya ringspot virus isolates among different population groups

| Groups | Fu and Li’s D | Fu and Li’s F | Tajima’s D |
|---|---|---|---|
| **PRSV-W** | | | |
| This study | -0.80ns | -1.12ns | -1.16ns |
| Americas | -0.90 ns | -0.97ns | -0.73ns |
| Oceania | -0.27ns | -0.44ns | -0.70ns |
| Asia | -0.49ns | -0.86ns | -1.19ns |
| Overall | -1.67ns | -1.98ns | -1.63 ns |
| **PRSV P&W** | | | |
| Americas | -1.06ns | -1.29ns | -1.20ns |
| Asia | -0.71ns | -1.13ns | -1.28ns |
| PRSVP | -0.51ns | -0.72ns | -0.75ns |
| Overall | -1.53ns | -1.81ns | -1.50ns |

ns; not significant, *;0.02<P<0.05, **; P<0.02
